# Effect and Mechanism of an ACT‐Based Psychological Resilience Intervention Targeting Students Failing in Postgraduate Entrance Examinations in China: A Randomized Controlled Trial

**DOI:** 10.1002/pchj.825

**Published:** 2025-01-08

**Authors:** Jing Han, Ziyi Zhao, Zhihong Ren

**Affiliations:** ^1^ Key Laboratory of Adolescent Cyberpsychology and Behavior (Ministry of Education), Key Laboratory of Human Development and Mental Health of Hubei Province, National Intelligent Society Governance Experiment Base (Education), School of Psychology Central China Normal University Wuhan China; ^2^ School of Education Science at Nanyang Normal University Nanyang China

**Keywords:** acceptance and commitment therapy, online intervention, psychological distress, psychological resilience

## Abstract

The postgraduate entrance examination frenzy is a widespread and intense phenomenon in China. As the number of students who failed the examination surged, the ensuing mental health problems became prominent. This study aimed to evaluate the effectiveness of an online ACT‐based group resilience course, which incorporated the six core components of ACT and integrated elements of Chinese culture, in alleviating psychological distress among students who failed the examination. It also explored the mechanism by which the intervention affected changes in psychological distress. A total of 61 participants were randomly assigned to the intervention group (*n* = 31) and the control group (*n* = 30). They attended an 8‐day group course, with 2‐h sessions each day. The study outcomes were psychological distress, resilience, psychological flexibility (PF), and psychological inflexibility (PI). These outcomes were measured at baseline, post‐intervention, and 1‐month follow‐up. Linear mixed models (LMMs) showed significant group × time interactions for all outcome variables, except for stress, which showed a marginally significant interaction. Post hoc analyses revealed significant improvements in depression, resilience, and PF at both post‐intervention and at the 1‐month follow‐up. Additionally, significant reductions in anxiety and a marginally significant reduction in stress were observed at the 1‐month follow‐up. However, no significant reduction was found in PI. The multiple mediation model showed that the intervention improved psychological distress by increasing resilience and PF. These findings suggest that online resilience group intervention is generally effective in enhancing resilience and alleviating psychological distress and is acceptable to students who have failed the postgraduate entrance examination, as evidenced by high participant engagement and satisfaction.

## 1 Introduction

In recent years, with the popularization of higher education, the number of Chinese university graduates has steadily increased. In 2022, the number of university graduates reached a record high of 10.76 million, the largest increase in decades (Ministry of Education of the People's Republic of China 2021). However, the mismatch between the traditional model of talent cultivation model and the diverse demands of contemporary society, along with the economic recession caused by the COVID‐19 pandemic, has worsened the employment challenges faced by university graduates (Li 2020). This has led to the growing phenomenon of involution in China. Furthermore, the entrenched exam‐oriented mindset, the excessive reverence for higher education, and the aspiration to achieve social mobility through education have collectively intensified academic anxiety (Hou and Zhao 2023). As a result, an increasing number of university students are opting for postgraduate studies to alleviate this pressure, making postgraduate entrance exam candidates a distinct and prominent group on campus.

Research by Ang, Shorey, et al. (2022), and Gong and Tian (2023) indicates that contemporary university students generally show high self‐esteem, overconfidence, and a reluctance to seek help when facing adversity. Students preparing for the postgraduate entrance examination often exhibit traits of stubbornness. This combination of high self‐esteem, stubbornness, and negative coping mechanisms in the face of adversity often leads to increased psychological inflexibility (PI) and heightened vulnerability among these candidates.

In 2022, 4.57 million candidates took the postgraduate entrance exam in China, with 1.242 million getting admitted, resulting in an admission rate of 27% (National Bureau of Statistics 2023). The low admission rate leaves many candidates facing the prospect of failure, which triggers a self‐identity crisis, disrupts career planning, and creates a role vacuum (where the individual is neither able to maintain their student status nor to prepare for employment) (Chen and Tang 2023). Failure also induces feelings of guilt toward family, increases financial pressure (Sun 2023), and fosters avoidance behaviors (Cao, Zhu, and Meng 2021). These factors contribute to psychological issues like depression, anxiety (Chen and Tang 2023; Xu 2014), decreased self‐efficacy, avoidance of past events and future prospects (Cheng 2020), and stress (Wang 2022). The postgraduate entrance examination frenzy is a unique phenomenon in China, and, as such, there is limited relevant research abroad. However, foreign research on exam failure has yielded similar conclusions, such as the shame of failure (Lam 2017), pessimism, and low self‐efficacy (Kruger et al. 2016).

Additionally, research has shown that failure in high‐stakes exams not only causes psychological distress but also contributes to subsequent academic failure (Beck et al. 2024).

Although failure in postgraduate entrance exam can be a significant blow, not all individuals experience lasting psychological distress. Some may navigate adversity with little or no long‐term effects. The concept of resilience offers a possible explanation for how these individuals maintain positive mental health in the face of challenges (Masten 2001). Resilience refers to positive adaptation in the face of significant challenges, encompassing successful life‐course development during or after exposure to potentially life‐altering experiences (Masten et al. 2009). Current resilience literature views resilience as a set of capabilities that can be developed through training (Alvord, Rich, and Berghorst 2016). Most resilience interventions focus on strengthening protective factors, such as individual characteristics and social support (Windle, Bennett, and Noyes 2011). A large body of research supports the effectiveness of these interventions in enhancing resilience and improving mental health (Alvord, Rich, and Berghorst 2016; Dray et al. 2017; Hjemdal et al. 2011).

To date, with the emergence of the “third wave” of psychotherapies, many resilience interventions have been based on acceptance and commitment therapy (ACT; Hayes, Strosahl, and Wilson 2011). ACT consists of six core processes: acceptance, cognitive defusion, contact with the present moment, self‐as‐context, value, and committed action, which aim to enhance psychological flexibility (PF). PF refers to acting by one's chosen values even when facing undesired internal experiences (Bond et al. 2011). This concept is closely tied to resilience, as PF is a key protective factor that enables individuals to persist with important developmental tasks during adversity (Ernst and Mellon 2016; Bryan, Ray‐Sannerud, and Heron 2015). Research on university students underscores the significance of value‐based action, goal setting, and emotional regulation in rebuilding psychological resilience (Ang, Lau, et al. 2022; Ceary, Donahue, and Shaffer 2019; Pan 2011). These elements are closely linked to the core concept of PF in ACT. Furthermore, ACT incorporates theoretical and practical techniques that are rooted in Eastern cultural traditions and philosophies, particularly Buddhism and Zen Buddhism. It aligns with Eastern cultural values (Fung 2015) and shares similarities with Chinese Daoist thought (He et al. 2017). Consequently, ACT‐based resilience interventions are particularly suitable in the Chinese context.

However, to the best of our knowledge, while there have been ACT interventions for university students (Howell and Passmore 2019), there is a notable lack of ACT‐based resilience interventions specifically for individuals who have failed postgraduate entrance exams. Given the increasing number of individuals in this group, it is crucial to alleviate their psychological stress and rebuild their resilience due to their PI and the unique internal and external factors contributing to their psychological vulnerability. Such interventions not only enhance their individual well‐being but also reduce the broader societal and national burden associated with psychological distress.

The COVID‐19 pandemic has transformed mental health service delivery, increasing the demand for online interventions due to restrictions on face‐to‐face interactions (King, Spencer, and Meeks 2021). Internet‐based ACT interventions have demonstrated effectiveness in enhancing resilience and improving mental health outcomes (Brown et al. 2016; Vahabi et al. 2022). In response, we developed and delivered an 8‐day ACT‐based resilience group course via online video conferencing. This delivery format not only ensured the continuity of intervention during the pandemic but also fostered greater interaction and mutual support among participants, both of which were crucial for building resilience. Additionally, the study assessed the effectiveness and acceptability of this program.

ACT aims to cultivate PF while overcoming PI. Although PF and PI are related constructs (Rogge et al. 2019; Rolffs, Rogge, and Wilson 2018), they are distinct and can change independently (Daks, Peltz, and Rogge 2020). These dimensions can coexist to varying degrees within individuals and independently affect mental health outcomes (Stabbe, Rolffs, and Rogge 2019). Research has demonstrated that higher PF is generally associated with better mental health and greater resilience, whereas higher PI is associated with more avoidant behaviors, lower resilience, and increased psychological distress (Bond et al. 2011; Waters et al. 2018; Howell and Demuynck 2021). Resilience, on the other hand, has been shown to enhance mental health and well‐being. In light of these findings, the present study aims to investigate whether the intervention leads to improved resilience, enhanced PF, and reduced PI. Furthermore, we seek to examine whether resilience, PF, and PI mediate improvement in psychological distress and whether resilience independently contributes to the improvement in psychological distress, beyond the mediating effects of PF and PI, to assess the key factors contributing to the effectiveness of resilience interventions.

In summary, the primary aim of the present study is to explore the effectiveness of this program. We hypothesize that the intervention will enhance psychological resilience and reduce psychological distress, accompanied by increased PF and decreased PI. Additionally, the secondary aim is to investigate the underlying mechanisms through which the intervention exerts its effects. We hypothesize that the changes in psychological distress will be mediated by improvements in resilience, PF, and reductions in PI.

## 2 Methods

### 2.1 Participant Recruitment and Selection

Advertisements for an online group course were posted on WeChat, the most widely used social media platform in China, and on Exam Forums, an online platform providing various resources for postgraduate entrance examination candidates. The program aimed to recruit students nationwide who had experienced failure in the postgraduate entrance exams and lacked sufficient psychological support. Recruitment was opened for 3 weeks following the graduate examination interview in May 2022, with the intervention conducted in early June. All recruited participants underwent an online group interview conducted by a psychologist with clinical experience, who assessed their eligibility for the study. Those meeting the inclusion criteria were subsequently invited to participate in the group course.

**FIGURE 1:**
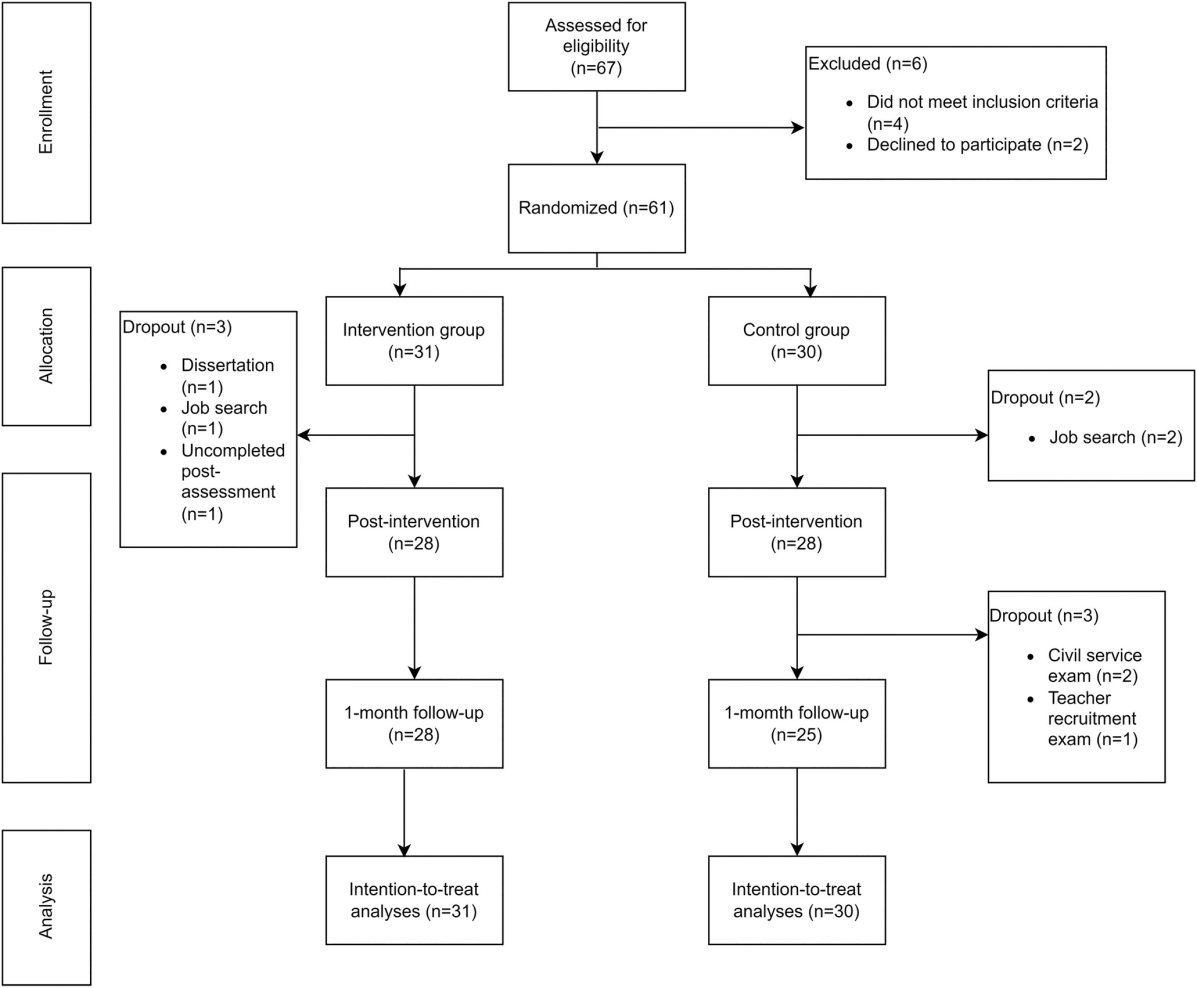
CONSORT flowchart of the study design.

The inclusion criteria were as follows: (1) having failed the postgraduate entrance examination in 2022; (2) being able to understand the research objectives and the intervention plan; (3) having access to the internet; and (4) voluntarily committing to full participation in the eight‐day intervention. To maximize participation inclusion, we did not conduct a clinical assessment of the psychological distress of the participants but rather relied on their self‐report of psychological distress. Exclusion criteria included: (1) self‐reported history of mental illness; (2) suicidal tendencies (score ≥ 7 on the Suicide Behavior Questionnaire‐Revised; Osman et al. 2001); and (3) receiving other psychotherapy or taking antidepressants within 3 months and other psychotropic drugs. Further details on participant recruitment are presented in the CONSORT flowchart depicted in Figure 1.

### 2.2 Procedure

Prior to obtaining consent, eligible participants were informed about the potential benefits and risks of the study and their rights and obligations. Written informed consent was obtained from all participants before intervention. An independent researcher who was not working on this program randomly assigned (1:1) the participants to either the intervention group or the control group using a free random sequence generator (www.random.org). The study employed a single‐blind parallel‐group design, with randomization information withheld from participants until the intervention was assigned. All participants completed a series of online assessments via the Questionnaire Star platform during the week before starting the program (pre‐assessment), the week after its completion (post‐assessment), and 1 month later (follow‐up assessment). At the end of the last assessment session, each participant received a report summarizing changes in his or her questionnaire measures. The program was conducted via Tencent Conference, an application enabling users to host or join meetings anytime, anywhere. It was approved by the ethics committee of Central China Normal University (CCNU‐IRB‐202207001) and was preregistered at osf.io (https://osf.io/pqj67).

### 2.3 Measures

#### 2.3.1 Demographic Questionnaire

Demographic Characteristics, including gender, major, family situation, and educational institution, were obtained at baseline.

#### 2.3.2 Depression Anxiety Stress Scale (DASS‐21)

Psychological distress over the previous week was assessed using the short version of the Depression Anxiety Stress Scale (DASS‐21) (Lovibond and Lovibond 1995). The scale consists of 21 items, and higher scores indicate higher distress. The internal consistency scores of the Chinese version of the depression, anxiety, and stress subscales are 0.77, 0.79, and 0.76, respectively, and the total scale is 0.89 (Gong et al. 2010). In the present study, the internal consistency was *α*_depression_ = 0.88, *α*_anxiety_ = 0.83, *α*_stress_ = 0.87, *α*_total_ = 0.94.

#### 2.3.3 Connor–Davidson Resilience Scale (CD‐RISC‐10)

Psychological resilience was measured using the 10‐item Connor–Davidson Resilience Scale (CD‐RISC‐10; Campbell‐Sills and Stein 2007). The scale consists of 10 items scored on a Likert scale of 1–5, with higher scores representing stronger psychological resilience. The scale has demonstrated good internal consistency in Chinese culture (Cronbach’ *α* = 0.74). (Zhang, Xiong, and Li 2018), and in the present study, the internal consistency was *α* = 0.92.

#### 2.3.4 The Multidimensional PF Inventory (MPFI‐24)

PF and PI were measured using the multidimensional psychological flexibility inventory (MPFI‐24). The multidimensional psychological flexibility inventory (Landi et al. 2021) aims to measure global PF and inflexibility. It consists of two subscales, flexibility (MPFI‐F) and inflexibility (MPFI‐I), measuring the six dimensions of flexibility and the six corresponding dimensions of inflexibility within the Hexaflex model. Participants responded to 24 items on a 6‐point Likert scale. The scale has demonstrated good internal consistency (Landi et al. 2021). Since there is no official Chinese version of MPFI, we translated it into Chinese following the procedures in scale translation outlined by Brislin (1970). In the present study, the internal consistency was *α*_flexibility_ = 0.90, *α*_inflexibility_ = 0.89.

#### 2.3.5 The Acceptance and Action Questionnaire‐II (AAQ‐II)

The Acceptance and Action Questionnaire‐II (AAQ‐II) (Bond et al. 2011) measures the PI. It consists of seven items on the scale, and a higher score indicates a higher degree of inflexibility. The Chinese version of AAQ‐II has excellent internal consistency (Cronbach’ *α* = 0.88) (Cao, Ji, and Zhu 2013). In the present study, the internal consistency was *α* = 0.74.

To distinguish between the two measures of PI, we refer to the PI measured by the MPFI as PI_MPFI‐I_, and that measured by the AAQ‐II as PI_AAQ‐II_.

#### 2.3.6 Acceptability and Dropout Analysis

To evaluate the acceptability of the intervention, participants were invited to respond to four open‐ended questions regarding their satisfaction with the intervention and suggestions for the future groups. Additionally, seven questions about their current state were rated on a 5‐point scale. The assessment was conducted solely in the intervention group. For the dropout assessment, the number of enrollees, attendees, and reasons for dropout were recorded (for details, see Appendix A).

### 2.4 Intervention

Participants in the intervention group engaged in an internet‐based resilience program grounded in ACT principles, incorporating elements of Chinese culture. This program was designed to address psychological challenges associated with examination failures, such as self‐doubt, uncertainty about future prospects, and familial and societal pressure. The intervention comprised an 8‐day course with daily 2‐h group training sessions, focusing on the following core components: (a) psychoeducation on psychological distress related to postgraduate entrance examination failure; (b) emotion regulation techniques; (c) cognitive defusion strategies; (d) values clarification and action‐oriented steps; and (e) fostering a support network through interpersonal interactions. The course incorporated mindfulness meditation, group discussions, experiential exercises, writing, drawing, and homework assignments. Except for the first session, each session began with a review of the previous topic and homework, followed by a brief mindfulness meditation exercise. Each session centered on a core concept of the ACT, integrating the specific concerns of students who had failed the postgraduate entrance examination in a manner consistent with their interests and cultural background. For example, drawing and writing were employed to facilitate better self‐expression; metaphors within ACT incorporated content familiar to the participants; meditation practices integrated both Tai Chi and traditional sitting meditation to align with participants' cultural traditions. Additionally, the program accounted for the differences between Chinese collectivist values and Western individualist values typically emphasized in ACT. While participants were encouraged to embrace their collectivist values, which emphasize cooperation and mutual support, they were also cautioned against rigid adherence to an interdependent self‐concept (Fung 2015). The dual approach aimed to foster resilience by balancing cultural values with the flexibility needed for psychological growth.

The intervention was primarily conducted by a psychologist who had attended systematic ACT training and had accumulated 800 h of face‐to‐face group counseling experience. For group discussions, members were randomly divided into four groups, each consisting of eight members, led by group facilitators who were graduate psychology students familiar with ACT and trained in the intervention manual used in this study. To maintain intervention fidelity, each group facilitator also adhered to a standardized group discussion process. After each session, the group leader and facilitators reviewed the entire process, addressed any inconsistencies in the intervention, and identified the focus and considerations for the subsequent discussion. For a detailed description of the course, see the Table B.1.

To ensure the program's effectiveness, expert consultation was used to evaluate and refine the program before the intervention. The Delphi expert consultation is an expert consensus‐based decision‐making method widely used in health service research (Hasson, Keeney, and McKenna 2000). Eight experts with professional knowledge and experience in the relevant fields were selected and participated in two rounds of anonymous evaluation of the intervention program. Evaluations were conducted using a 5‐point Likert scale. The revised program received a favorable expert evaluation, with a credible coefficient of authority (Cr = 0.84 [0.7–1.0]) and an acceptable coefficient of variation (CV = 0–0.12). For details, see the Appendix C.

Prior to the intervention, researchers randomly selected six students from the general population to simulate the entire intervention process. Once everything was ready, the formal intervention commenced. Participants assigned to the control group did not receive any contact during the intervention period, except for measurement sessions. After the final assessment, they were provided with a series of resilience intervention materials and monetary compensation. In contrast, participants in the intervention group did not receive monetary compensation.

## 3 Data Analysis

Statistical analyses were conducted using SPSS 25 and the PROCESS macro tool (version 4.1) (A. F. Hayes 2017). Chi‐square and independent *t*‐tests were employed to investigate differences in participant characteristics and pre‐assessment outcomes at baseline.

For the intention‐to‐treat principle, we assessed intervention effects for each outcome variable using LMMs with restricted maximum likelihood and compound symmetry as covariance type, resulting in a 2 (group: intervention, control) × 3 (time: baseline, post‐intervention, 1‐month follow‐up) fixed effects model, with random effects accounting for individual variation. The LMMs analysis was robust in handling missing data in the longitudinal study. The differential effectiveness of the intervention would be indicated by group‐by‐time interactions. Post hoc analyses were conducted to identify significant differences between measurements, with corrections for multiple testing using Bonferroni's correction. We calculated within‐group and between‐group effect sizes (*d*_Cohen's_) based on estimated means and the pooled standard deviations (Lakens 2013).

For the mediation analysis, residualized change scores were computed by regressing posttest scores on pretest scores and calculating the difference between observed and predicted posttest scores. These scores account for baseline differences, making them independent of a participant's pre‐intervention score (Cohen et al. 2002). Residualized change scores were computed for the mediator variables (resilience, PF, and PI), which showed significant improvements in the intervention group compared to the control group, as well as for the outcome variable (psychological distress). We then examined whether changes in resilience, PF, and PI (M) mediated the intervention's effectiveness (X) in terms of the change in psychological distress (Y). The multiple mediation analysis can help to determine to what extent the change in psychological distress brought by intervention is mediated by these variables and whether the mediation of a particular variable is independent of the other mediators (Knoop, Van Kessel, and Moss‐Morris 2012).

G*Power (Faul et al. 2007) version 3.1.9 was utilized to calculate the sample size. With an alpha value of 0.05 and a power of 0.95, we assumed an effect size (Cohen's *d*) of 0.48, based on the results of a previous meta‐analysis (Liu et al. 2020). The expected sample size was at least 30 participants per group. After data collection, we calculated the statistical power of our sample (*n* = 61) based on the recommendations for LMMs sample size provided by Kumle, Võ, and Draschkow (2021). Our analysis revealed a statistical power of 0.79, close to the conventional threshold of 0.80, indicating that our sample size is nearly adequate to meet the standard requirement for sufficient power.

## 4 Results

### 4.1 Participants' Baseline Characteristics

A total of 61 senior undergraduate students participated in the study, 31 in the intervention group and 30 in the control group. Two‐thirds of the participants (41/61, 67.2%) were female, and nearly half (27/61, 44.3%) were students majoring in natural sciences. Most participants came from two‐parent families (55/61, 90.2%) and many of them were from ordinary universities (48/61, 78.7%). At baseline comparison, no significant differences were observed in all demographic characteristics between groups, except for gender, with a higher proportion of female participants in the intervention group compared to the control group (*p* = 0.023) (see Table 1). Moreover, no significant differences were found in all outcomes (ps > 0.05) between groups.

**TABLE 1:** Demographic variables at baseline for participants in both groups.

	Intervention (*n* = 31) *n* (%) or M ± SD	Control (*n* = 30) *n* (%) or M ± SD	Test statistic
Gender			*χ* ^2^ (1) = 5.161, *p* = 0.023
Male	6 (30)	14 (70)	
Female	25 (61)	16 (39)	
Major			*χ* ^2^ (1) = 7.244, *p* = 0.065
Liberal arts	6 (19.4)	13 (43.3)	
Natural science	14 (45.2)	13 (43.3)	
Engineering	4 (12.9)	3 (10)	
Art	7 (22.6)	1 (3.3)	
Family situation			*χ* ^2^ (1) = 2.815, *p* = 0.093
Parents	26 (83.9)	29 (96.7)	
Single parent	5 (16.1)	1 (3.3)	
School			*χ* ^2^ (1) = 6.462, *p* = 0.091
World‐class university	5 (16.1)	0	
Key university	4 (12.9)	3 (10)	
Ordinary university	22 (71)	26 (86.7)	
College	0	1 (3.3)	

Abbreviations: *χ* ^2^, Pearson's chi‐square; M, mean; SD, standard deviation.

### 4.2 Acceptability and Dropout Analysis

Regarding acceptability, participants gave relatively high ratings for both intervention satisfaction and self‐status assessment. No harm was reported during the intervention. As for dropout, throughout the program period, three participants in the intervention group withdrew due to dissertation commitments, job search, and incomplete post‐assessment, while five participants in the control group dropped out due to job search, civil service exams, and teacher recruitment exams. There was no significant difference between the two groups. For more detailed information, see Appendix A.

### 4.3 Changes in Outcomes After Intervention

The effectiveness of intervention in improving resilience, psychological distress, PF, and PI was calculated using LMMs. All of outcomes were qualified by significant or marginally significant group × time interactions (DASS‐D: *F*_(2,105.25)_ = 9.41, *p <* 0.001; DASS‐A: *F*_(2,105.95)_ = 6.99, *p =* 0.001; DASS‐S: *F*_(2,106.30)_ = 2.95, *p* = 0.057; CD‐RISC: *F*_(2,105.90)_ = 8.27, *p* < 0.001; MPFI‐F: *F*_(2,105.02)_ = 9.60, *p* < 0.001; MPFI‐I: *F*_(2,108.52)_ = 4.48, *p =* 0.014; AAQ‐II: *F*_(2,106.98)_ = 4.219, *p =* 0.017). Post hoc analyses showed that there were significantly lower scores of DASS‐D in the intervention group at post‐intervention (*d = −*0.65, *p =* 0.013) and follow‐up period (*d* = −0.75, *p =* 0.007). The scores of DASS‐A and DASS‐S were not lower than the control group after intervention (*d = −*0.39, *p =* 0.133; *d = −*0.40, *p =* 0.127, respectively), but significantly or marginally significantly lower at the follow‐up period (*d = −*0.54, *p =* 0.04; *d = −*0.48, *p =* 0.065, respectively). For the CD‐RISC, post hoc analysis showed a significant difference between the two groups at post‐intervention (*d =* 0.58, *p =* 0.027) and follow‐up (*d =* 0.57, *p =* 0.031). For the MPFI‐F, post hoc analysis showed significantly higher scores in the intervention group at post‐intervention (*d =* 0.70, *p =* 0.008) and follow‐up period (*d =* 0.64, *p =* 0.016), However, there was no significant between‐group difference in MPFI‐I scores at post‐intervention (*d = −*0.40, *p =* 0.132) or follow‐up (*d = −*0.20, *p =* 0.435). For the AAQ‐II, post hoc analysis showed that the scores of the intervention group were lower than the control group after intervention (*d = −*0.59, *p =* 0.026), but no significant differences were registered at the follow‐up period (*d = −*0.21, *p =* 0.423).

All outcomes had significant within‐group differences in the intervention group, but no significant within‐group difference in the control group. Detailed information on means, standard errors, and effect sizes is provided in Table 2.

**TABLE 2:** Observed and estimated means of all outcome measures and within‐ and between‐group effect sizes.

	Pre‐assessment M (SD)	*n*	Post‐assessment (estimated) M (SE)	*n*	Follow‐up (estimated) M (SE)	*n*	Pre‐post within *d*_cohen's_ (CI)	Post between *d*_cohen's_ (CI)	Pre‐follow‐up within *d*_cohen's_ (CI)	Follow‐up between *d*_cohen's_ (CI)
CD‐RISD										
IG	23.10 (8.08)	31	29.74 (1.16)	31	29.67 (1.16)	31	**1.06 (0.53, 1.59)**	**0.58 (0.07, 1.10)**	**1.05 (0.52, 1.58)**	**0.57 (0.06, 1.08)**
CG	24.7 (5.89)	30	26.05 (1.17)	30	26.01 (1.21)	30	0.21 (−0.29, 0.72)		0.21 (−0.30, 0.71)	
DASS										
DASS‐D										
IG	6.23 (5.64)	31	2.30 (0.82)	31	2.15 (0.82)	31	**−0.90 (−1.42, −0.37)**	**−0.65 (−1.17, −0.14)**	**−0.93 (−1.45, −0.40)**	**−0.75 (−1.26, −0.23)**
CG	5.23 (4.88)	30	5.20 (0.82)	30	5.53 (0.85)	30	−0.008 (−0.51, 0.50)		0.06 (−0.44, 0.57)	
DASS‐A										
IG	5.94 (4.82)	31	2.86 (0.74)	31	2.40 (0.74)	31	**−0.77 (−1.28, −0.25)**	−0.39 (−0.90, 0.11)	**−0.88 (−1.40, −0.36)**	**−0.54 (−1.05, −0.03)**
CG	5.33 (3.72)	30	4.45 (0.75)	30	4.61 (0.76)	30	−0.22 (−0.73, 0.29)		−0.18 (−0.69, 0.33)	
DASS‐S										
IG	8.06 (5.12)	31	4.72 (0.90)	31	4.26 (0.90)	31	**−0.69 (−1.20, −0.17)**	−0.40 (−0.91, 0.11)	**−0.78 (−1.30, −0.26)**	−0.48 (−0.99, 0.02)
CG	8.10 (5.35)	30	6.69 (0.91)	30	6.68 (0.94)	30	−0.29 (−0.80, 0.22)		−0.29 (−0.80, 0.22)	
MPFI										
MPFI‐F										
IG	31.45 (11.21)	31	41.37 (1.81)	31	41.30 (1.81)	31	**1.02 (0.49, 1.55)**	**0.70 (0.18, 1.22)**	**1.01 (0.48, 1.54)**	**0.64 (0.12, 1.15)**
CG	34.43 (6.76)	30	34.49 (1.81)	30	34.89 (1.89)	30	0.006 (−0.50, 0.51)		0.05 (−0.46, 0.55)	
MPFI‐I										
IG	33.84 (9.66)	31	27.31 (2.15)	31	27.92 (2.15)	31	**−0.56 (−1.07, −0.05)**	−0.40 (−0.90, 0.11)	**−0.51 (−1.02, −0.004)**	−0.20 (−0.71, 0.30)
CG	30.73 (12.43)	30	31.95 (2.17)	30	30.35 (2.23)	30	0.11 (−0.40, 0.61)		−0.03 (−0.54, 0.47)	
AAQ‐II										
IG	25 (8.71)	31	21.09 (1.40)	31	21.69 (1.40)	31	**−0.52 (−1.02, −0.01)**	−0.59 (−1.10, −0.07)	−0.44 (−0.94, 0.07)	−0.21 (−0.71, 0.29)
CG	24.2 (8.24)	30	25.57 (1.41)	30	23.32 (1.46)	30	0.18 (−0.32, 0.69)		−0.13 (−0.64, 0.38)	

*Note: d*_cohen's_ based on estimated means and the pooled standard deviations; AAQ‐II, The Acceptance and Action Questionnaire‐II; CD‐RISC, Connor–Davidson Resilience Scale; CI, 95% confidence interval; DASS, Depression Anxiety Stress Scale; MPFI, The Multidimensional Psychological Flexibility Inventory. Significant differences are highlighted in bold.

Abbreviations: CG, control group; IG, intervention group; M, mean; SD, standard deviation; SE, standard error.

Since there was no significant improvement in the MPFI‐I in the intervention group compared to the control group after the intervention, the study further examined the differences between the intervention and control groups across the six dimensions of the MPFI‐I. It was found that the intervention group showed significant improvements in the dimensions of inattentive and unaware (*d = −*0.60, *p =* 0.036) and self‐as‐content (*d = −*1.02, *p =* 0.001), while there were no significant improvements in the dimensions of experiential avoidance (*d =* 0.38, *p =* 0.166), fusion (*d =* 0.25, *p =* 0.424), losing touch with values (*d = −*0.43, *p =* 0.419), and inaction (*d = −*0.29, *p =* 0.114). For details, see Table D.1.

### 4.4 Mediators of Change in Treatment Outcome

**FIGURE 2:**
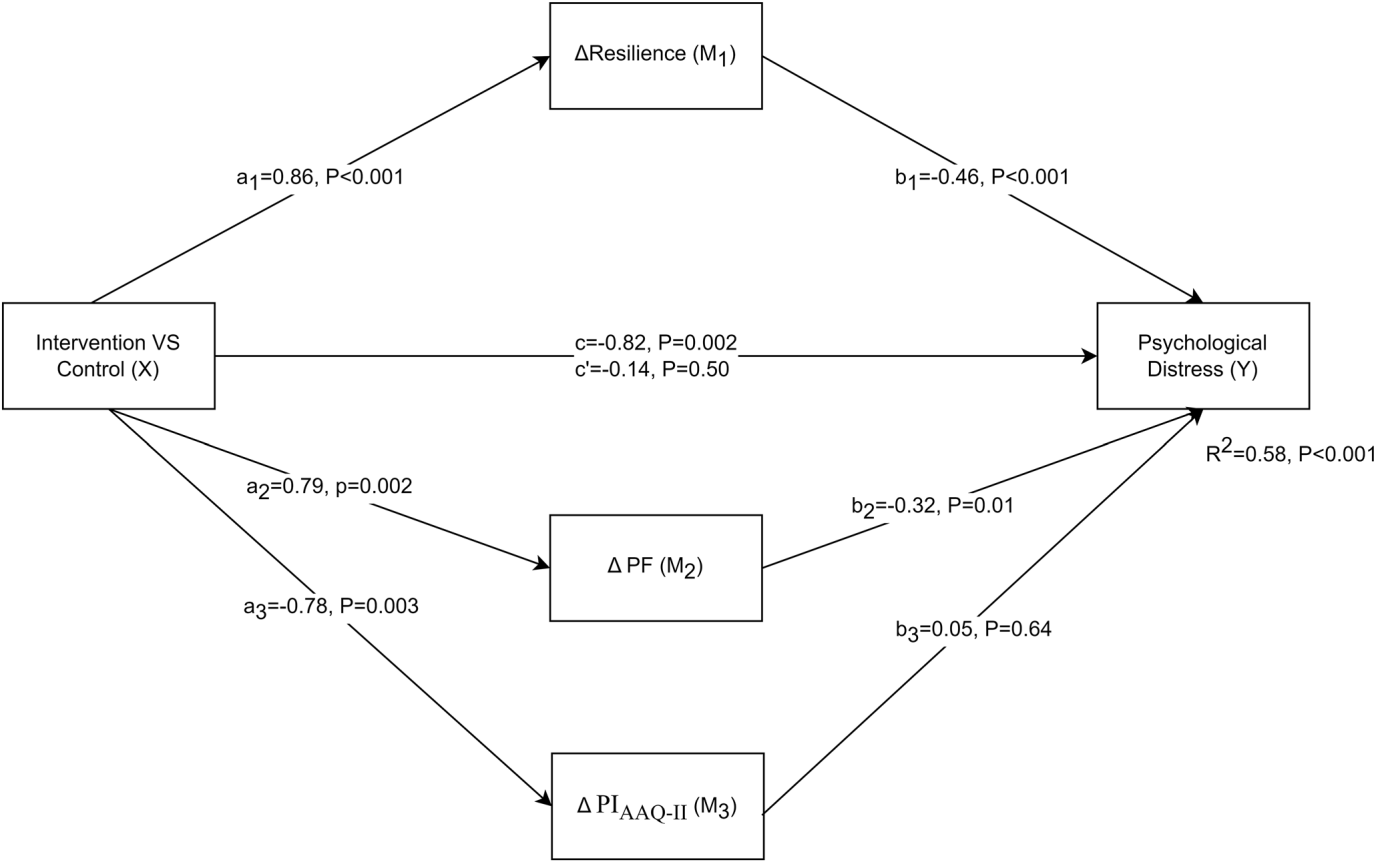
Multiple mediation model of Intervention Effect on Psychological Distress. PF, Psychological Flexibility; PI_AAQ‐II_, Psychological Inflexibility. which measured by AAQ‐II.

To investigate the mechanisms of change, the residualized change scores for resilience, PF, and PI_AAQ‐II_, which showed significant differences between the two groups, were entered into a multiple mediation analysis to prevent multicollinearity, stepwise regression was used to examine the impact of putative mediator variables on intervention outcomes. The multicollinearity test found that the tolerance of each model was greater than 0.2, and the variance inflation factor was less than 5, suggesting that multicollinearity was not a concern. Figure 2 shows the standardized coefficients for changes in psychological distress after intervention. The percentage of variance (*R* ^2^) explained by the differences between the two groups exceeded 58%. The a‐path was significant in resilience, PF, and PI_AAQ‐II_. The b‐path was significant in resilience, PF but not in PI_AAQ‐II_. The direct effect of the intervention on psychological distress was no longer significant; it was entirely mediated by resilience (ab = −0.39, s.e. = 0.13, 95% CI [−0.66, −0.13]) and PF (ab = −0.25, s.e. = 0.11, 95% CI [−0.49, −0.05]).

## 5 Discussion

This study explored the effectiveness of an intervention aimed at helping students who had failed the postgraduate entrance examination to improve their psychological resilience and alleviate their psychological distress. The results demonstrated a significant alleviation in depression and a notable increase in resilience within the intervention group. Although there was no significant decrease in anxiety and stress immediately after the intervention, anxiety was significantly reduced during the one‐month follow‐up period, and stress demonstrated a marginally significant reduction. PF and PI_AAQ‐II_ demonstrated significant improvements; however, PI_MPFI‐I_ did not exhibit a significant reduction. Resilience and PF were found to mediate the mitigation of psychological distress. The intervention had a low attrition rate and high acceptability.

The study found that the intervention group exhibited significant improvements in resilience, with medium to large effect sizes at both post‐intervention and 1‐month follow‐up compared to the control group. These results suggested that our program was effective. These findings were consistent with improvements shown in previous resilience studies with parentally bereaved college students (Murrell et al. 2018), stressed college students (Steinhardt and Dolbier 2008), and children and adolescents (Dray et al. 2017). Specifically, this study demonstrated that resilience interventions for students who had failed the postgraduate entrance examination were effective in alleviating their unique psychological distress. A key strength of the intervention lies in its integration of Chinese cultural elements—such as Tai Chi, traditional sitting meditation, and collectivist values—which enhanced its cultural relevance and participant engagement. The integration of ACT principles with culturally familiar practices strengthened the intervention's applicability in the Chinese context and facilitated the broader cross‐cultural expansion of ACT. These findings align with research by Chinese scholars (He et al. 2017; Fang et al. 2022), further supporting the cultural adaptation of ACT in China. Additionally, the use of an internet‐based intervention proved feasible, making it a suitable option in the context of public health emergencies such as the COVID‐19 pandemic. This finding was further supported by a meta‐analysis of iACT (Thompson et al. 2021).

The present study demonstrated that the intervention group showed a significant reduction in depression at both post‐intervention and 1‐month follow‐up, with medium to large effect sizes, and a significant/marginally significant reduction in anxiety and stress at 1‐month follow‐up, with small to medium effect sizes. This result supported a meta‐analysis of iACT, which mentioned that non‐clinical samples had less room to reduce psychological symptoms except for depression (Thompson et al. 2021). In addition, after taking the postgraduate entrance examination, participants faced different choices such as whether to seek employment or continue to prepare for the postgraduate exam, each of which was challenging. Hence, the stress felt by them persisted. Moreover, the ongoing COVID‐19 pandemic led to a lasting negative impact on mental health among all college students (Husky et al. 2020; Son et al. 2020). It may help explain the lack of significant alleviation in stress and anxiety immediately after the intervention. Also, the intervention only lasted 8 days, although internet‐based interventions are often shorter than face‐to‐face interventions (Brog et al. 2022). Participants may need more time to practice and internalize, so the effect is delayed (Bastounis et al. 2016). It may be another reason for the absence of immediate significant differences in stress and anxiety. Indeed, future research with larger sample sizes will be necessary to validate these trends. Moreover, resilience interventions should be further strengthened through continuous optimization of training programs and by allocating more time for reflection.

In terms of PF and PI, compared to the control group, the intervention group showed significant improvements in PF at both time points, while the results for PI (PI_MPFI‐I_ and PI_AAQ‐II_) were inconsistent. Further testing revealed that the dimensions of “inattentive and unaware” and “self‐as‐content” dropped significantly after the intervention. In contrast, the dimensions of “experiential avoidance,” “fusion,” “losing touch with values,” and “inaction” did not show significant decreases. These findings were inconsistent with our hypothesis that both PF and PI would exhibit significant changes. On the one hand, this may indicate that certain dimensions of PI are more resistant to change in the group that experienced failure in postgraduate entrance exams. On the other hand, the inconsistencies in PI measurements suggest that unidimensional measures of PI may not be sufficient to capture the six subdimensions and are not sensitive to specific subdimensions, supporting the views of Rolffs, Rogge, and Wilson (2018).

We attempt to further elucidate these results. First, the lack of PF can be divided into three processes: the first involves low mindfulness, the second reflects a lack of openness to experience, and the third concerns a lack of clarity and commitment to personal values (Glick, Millstein, and Orsillo 2014; Morris and Mansell 2018; Kashdan and Rottenberg 2010; Rolffs, Rogge, and Wilson 2018; Kashdan et al. 2020). Our intervention improved low mindfulness but did not improve experiential avoidance, which is inconsistent with previous research primarily focused on Western populations (Aguirre‐Camacho et al. 2017; Brown, Whittingham, and Sofronoff 2015). These studies have shown that, in Western countries, experiential avoidance indicates low functioning, whereas, in Eastern countries such as China, experiential avoidance may be associated with higher functioning, representing an adaptive response that meets social expectations (Lin et al. 2020). Thus, for Chinese students, this coping style is more ingrained and culturally adapted. Furthermore, in collectivist cultures, value clarification can be particularly challenging. Chinese culture emphasizes the alignment of individual values with collective and social values. The pressure to conform to collective values may overshadow the pursuit of individual values, making it difficult to distinguish between personal and group values (Vauclair et al. 2015). This lack of clarity in individual values can further contribute to the inability to act independently. Second, prior research has shown that high PI is positively associated with stress, worry, and anxiety (Tavakoli et al. 2019). Due to the ongoing real‐world pressures faced by students who have failed postgraduate entrance exams (Gong and Tian 2023), achieving significant changes in PI is particularly challenging. Third, Pakenham (2015)'s meta‐analysis of online interventions indicated that the effectiveness of the intervention was positively correlated with the frequency of intervention sessions. Thus, the duration of current interventions may not be sufficient to have a significant effect on some components of PI. Of course, these results must be interpreted with caution and validated by further research. In the meantime, these findings suggest a focus for future interventions.

Mechanisms of intervention studies have shown that resilience and PF mediate the reduction of psychological distress. Resilience and PF have varying predictive powers for psychological distress, with their combined effect being additive rather than overlapping. While the resilience intervention improved PF, this, in turn, helped reduce psychological distress. These results are consistent with previous studies (Pakenham et al. 2018; Giovannetti, Solari, and Pakenham 2022). However, resilience itself encompasses a broader range of elements. For instance, beyond the individual level, resilience also includes an individual's social competence. As supported by participant feedback, our group intervention helped foster interpersonal support and enhance social skills, both of which are protective factors against the negative effects of stressors (Southwick and Charney 2012). Moreover, as one meta‐analysis pointed out (Ang, Lau, et al. 2022), at the community level, schools can foster resilience through various activities. Especially in China, where collectivism is emphasized, faith, beliefs, and positive traditional culture play a crucial role in safeguarding students. All these factors should be considered in the optimization of future research programs.

Therefore, future research should explore more effective intervention strategies. For example, while acknowledging the cultural adaptability of experiential avoidance, participants could be encouraged to adopt more flexible coping strategies. More structured guidance in value clarification should be provided to help balance personal and collective values. Additionally, increasing the frequency and duration of intervention sessions may address the persistent challenges of PI. Furthermore, integrating support from schools and society could offer external guidance and assistance to students who have failed postgraduate entrance exams. Through continuous optimization of these interventions via multiple approaches, more effective support can be provided to help these students overcome their difficulties.

This study presents several limitations. First, due to the small sample size of the study, the findings need to be repeated for validation. Second, to encourage broader participation, this study did not establish a threshold for clinical distress, warranting future investigations to compare the benefits of intervention among subjects with varying levels of clinical distress. Third, although the entire intervention process aimed to ensure fidelity, no formal fidelity assessment was conducted, which will need to be addressed in future research. Fourth, while internet‐based group sessions eliminate spatial constraints, their time requirements and the need for open interaction may result in varying levels of participant engagement. Future research should explore flexible and diverse formats, such as incorporating self‐help modules or asynchronous sessions. Additionally, tailored and efficient services should be provided to meet the needs of participants with varying levels of psychological distress. For example, structured and frequent group interventions may be more suitable for participants with high levels of distress, while autonomous and flexible self‐help interventions could better support those with lower levels of distress.

## 6 Conclusion

The study is a preliminary exploration of resilience interventions for students who failed the postgraduate entrance exams. The intervention proved effective in reducing depression, enhancing resilience, and increasing PF among participants. However, significant or marginally significant improvements in anxiety and stress were observed only during the follow‐up period, while PI showed no significant improvement. Mechanistic analysis identified resilience and PF as key predictors of reduced psychological distress. Future resilience interventions henceforth should aim to expand the target population and provide more effective and tailored support.

## Ethics Statement

This study was approved by the Central China Normal University's ethics committee (CCNU‐IRB‐202207001) and pre‐registered at osf.io (https://osf.io/pqj67).

## Consent

Informed consent was obtained from all participants.

## Conflicts of Interest

The authors declare no conflicts of interest.

## Supporting information


Data S1.

## Data Availability

Data can be provided if required.

## References

[pchj825-bib-0001] Aguirre‐Camacho, A. , G. Pelletier , A. González‐Márquez , L. M. Blanco‐Donoso , P. García‐Borreguero , and B. Moreno‐Jiménez . 2017. “The Relevance of Experiential Avoidance in Breast Cancer Distress: Insights From a Psychological Group Intervention.” Psycho‐Oncology 26, no. 4: 469–475. 10.1002/pon.4162. 27228257

[pchj825-bib-0002] Alvord, M. K. , B. A. Rich , and L. H. Berghorst . 2016. “Resilience Interventions.” In APA Handbook of Clinical Psychology: Psychopathology and Health, edited by J. C. Norcross , G. R. VandenBos , D. K. Freedheim , and N. Pole , 505–519. Washington, DC: American Psychological Association.

[pchj825-bib-0003] Ang, W. H. D. , S. T. Lau , L. J. Cheng , et al. 2022. “Effectiveness of Resilience Interventions for Higher Education Students: A Meta‐Analysis and Metaregression.” Journal of Educational Psychology 114, no. 7: 1670–1694. 10.1037/edu0000719.

[pchj825-bib-0004] Ang, W. H. D. , S. Shorey , V. Lopez , H. S. J. Chew , and Y. Lau . 2022. “Generation Z Undergraduate Students' Resilience During the COVID‐19 Pandemic: A Qualitative Study.” Current Psychology 41, no. 11: 8132–8146. 10.1007/s12144-021-01830-4. 34253948 PMC8264489

[pchj825-bib-0077] Bastounis, A. , P. Callaghan , A. Banerjee , and M. Michail . 2016. “The Effectiveness of the Penn Resiliency Programme (PRP) and its Adapted Versions in Reducing Depression and Anxiety and Improving Explanatory Style: A Systematic Review and Meta‐analysis.” Journal of Adolescence 52: 37–48. 10.1016/j.adolescence.2016.07.004. 27494740

[pchj825-bib-0005] Beck, K. C. , H. L. Røhr , B. Reme , and M. Flatø . 2024. “Distressing Testing: A Propensity Score Analysis of High‐Stakes Exam Failure and Mental Health.” Child Development 95, no. 1: 242–260. 10.1111/cdev.13985. 37566438

[pchj825-bib-0006] Bond, F. W. , S. C. Hayes , R. A. Baer , et al. 2011. “Preliminary Psychometric Properties of the Acceptance and Action Questionnaire–II: A Revised Measure of Psychological Inflexibility and Experiential Avoidance.” Behavior Therapy 42, no. 4: 676–688. 10.1016/j.beth.2011.03.007. 22035996

[pchj825-bib-0007] Brislin, R. W. 1970. “Back‐Translation for Cross‐Cultural Research.” Journal of Cross‐Cultural Psychology 1: 185–216. 10.1177/135910457000100301.

[pchj825-bib-0008] Brog, N. A. , J. K. Hegy , T. Berger , and H. Znoj . 2022. “Effects of an Internet‐Based Self‐Help Intervention for Psychological Distress Due to COVID‐19: Results of a Randomized Controlled Trial.” Internet Interventions 27: 100492. 10.1016/j.invent.2021.100492. 34956841 PMC8684052

[pchj825-bib-0009] Brown, F. L. , K. Whittingham , and K. Sofronoff . 2015. “Parental Experiential Avoidance as a Potential Mechanism of Change in a Parenting Intervention for Parents of Children With Pediatric Acquired Brain Injury.” Journal of Pediatric Psychology 40, no. 4: 464–474. 10.1093/jpepsy/jsu109. 25535318

[pchj825-bib-0010] Brown, M. , A. Glendenning , A. E. Hoon , and A. John . 2016. “Effectiveness of Web‐Delivered Acceptance and Commitment Therapy in Relation to Mental Health and Well‐Being: A Systematic Review and Meta‐Analysis.” Journal of Medical Internet Research 18, no. 8: e6200. 10.2196/jmir.6200. PMC5039035 27558740

[pchj825-bib-0011] Bryan, C. J. , B. Ray‐Sannerud , and E. A. Heron . 2015. “Psychological Flexibility as a Dimension of Resilience for Posttraumatic Stress, Depression, and Risk for Suicidal Ideation Among Air Force Personnel.” Journal of Contextual Behavioral Science 4, no. 4: 263–268. 10.1016/j.jcbs.2015.10.002.

[pchj825-bib-0012] Campbell‐Sills, L. , and M. B. Stein . 2007. “Psychometric Analysis and Refinement of the Connor‐Davidson Resilience Scale (CD‐RISC): Validation of a 10‐Item Measure of Resilience.” Journal of Traumatic Stress 20, no. 6: 1019–1028. 10.1002/jts.20271. 18157881

[pchj825-bib-0013] Cao, C. , C. Zhu , and Q. Meng . 2021. “Chinese International Students' Coping Strategies, Social Support Resources in Response to Academic Stressors: Does Heritage Culture or Host Context Matter?” Current Psychology 40, no. 1: 242–252. 10.1007/s12144-018-9929-0.

[pchj825-bib-0014] Cao, J. , Y. Ji , and Z. H. Zhu . 2013. “Reliability and Validity of the Chinese Version of the Acceptance and Action Questionnaire‐Second Edition (AAQ‐II) in College Students.” Chinese Mental Health Journal 27, no. 11: 873–877.

[pchj825-bib-0015] Ceary, C. D. , J. J. Donahue , and K. Shaffer . 2019. “The Strength of Pursuing Your Values: Valued Living as a Path to Resilience Among College Students.” Stress and Health 35, no. 4: 532–541. 10.1002/smi.2886. 31276290

[pchj825-bib-0016] Chen, Z. , and X. Tang . 2023. “Constraints and Breakthroughs Under Postgraduate Entrance Exam Failure: A Narrative Study Based on Life Course Theory.” Education and Teaching Research 37, no. 10: 14–27. 10.13627/j.cnki.cdjy.2023.10.001.

[pchj825-bib-0017] Cheng, C. X. 2020. “A Study of Interpretative Phenomenological Psychology on the Experience of Failure in Postgraduate Entrance Examination,” Master's Dissertation, Nanjing Normal University. https://kns.cnki.net/KCMS/detail/detail.aspx?dbname=CMFD202101&filename=1021517927.nh.

[pchj825-bib-0018] Cohen, J. , P. Cohen , S. G. West , and L. S. Aiken . 2002. Applied Multiple Regression/Correlation Analysis for the Behavioral Sciences. 3rd ed. New York: Routledge.

[pchj825-bib-0019] Daks, J. S. , J. S. Peltz , and R. D. Rogge . 2020. “Psychological Flexibility and Inflexibility as Sources of Resiliency and Risk During a Pandemic: Modeling the Cascade of COVID‐19 Stress on Family Systems With a Contextual Behavioral Science Lens.” Journal of Contextual Behavioral Science 18: 16–27. 10.1016/j.jcbs.2020.08.003. 32834972 PMC7428754

[pchj825-bib-0020] Dray, J. , J. Bowman , E. Campbell , et al. 2017. “Systematic Review of Universal Resilience‐Focused Interventions Targeting Child and Adolescent Mental Health in the School Setting.” Journal of the American Academy of Child and Adolescent Psychiatry 56, no. 10: 813–824. 10.1016/j.jaac.2017.07.780. 28942803

[pchj825-bib-0022] Ernst, M. M. , and M. W. Mellon . 2016. “Acceptance and Commitment Therapy (ACT) to Foster Resilience in Pediatric Chronic Illness.” In Child and Adolescent Resilience Within Medical Contexts: Integrating Research and Practice, edited by C. DeMichelis and M. Ferrari, 193–207. Switzerland: Springer International Publishing. 10.1007/978-3-319-32223-0_11.

[pchj825-bib-0023] Fang, S. , D. Ding , P. Ji , M. Huang , and K. Hu . 2022. “Cognitive Defusion and Psychological Flexibility Predict Negative Body Image in the Chinese College Students: Evidence From Acceptance and Commitment Therapy.” International Journal of Environmental Research and Public Health 19, no. 24: 16519. 10.3390/ijerph192416519. 36554399 PMC9778665

[pchj825-bib-0024] Faul, F. , E. Erdfelder , A. G. Lang , and A. Buchner . 2007. “G*Power 3: A Flexible Statistical Power Analysis Program for the Social, Behavioral, and Biomedical Sciences.” Behavior Research Methods 39, no. 2: 175–191. 10.3758/BF03193146. 17695343

[pchj825-bib-0025] Fung, K. 2015. “Acceptance and Commitment Therapy: Western Adoption of Buddhist Tenets?” Transcultural Psychiatry 52, no. 4: 561–576. 10.1177/1363461514537544. 25085722

[pchj825-bib-0026] Giovannetti, A. M. , A. Solari , and K. I. Pakenham . 2022. “Effectiveness of a Group Resilience Intervention for People With Multiple Sclerosis Delivered via Frontline Services.” Disability and Rehabilitation 44, no. 22: 6582–6592. 10.1080/09638288.2021.1960441. 34406895

[pchj825-bib-0027] Glick, D. M. , D. J. Millstein , and S. M. Orsillo . 2014. “A Preliminary Investigation of the Role of Psychological Inflexibility in Academic Procrastination.” Journal of Contextual Behavioral Science 3, no. 2: 81–88. 10.1016/j.jcbs.2014.04.002.

[pchj825-bib-0028] Gong, H. , and H. Tian . 2023. “Entering the Postgraduate Entrance Exam Cycle: Examining College Students' Decision‐Making Behavior From a Bounded Rationality Perspective.” Academic Degrees and Graduate Education 7: 54–61. 10.16750/j.adge.2023.07.009.

[pchj825-bib-0029] Gong, X. , X. Y. Xie , R. Xu , and Y. J. Luo . 2010. “Psychometric Properties of the Chinese Versions of DASS‐21 in Chinese College Students. Chinese.” Journal of Clinical Psychology 18, no. 4: 443–446. 10.16128/j.cnki.1005-3611.2010.04.020.

[pchj825-bib-0030] Hasson, F. , S. Keeney , and H. McKenna . 2000. “Research Guidelines for the Delphi Survey Technique.” Journal of Advanced Nursing 32, no. 4: 1008–1015. 10.1046/j.1365-2648.2000.t01-1-01567.x. 11095242

[pchj825-bib-0031] Hayes, A. F. 2017. Introduction to Mediation, Moderation, and Conditional Process Analysis: A Regression‐Based Approach. New York: Guilford Publications.

[pchj825-bib-0032] Hayes, S. C. , K. D. Strosahl , and K. G. Wilson . 2011. Acceptance and Commitment Therapy: The Process and Practice of Mindful Change. New York: Guilford Press.

[pchj825-bib-0033] He, H. J. , M. R. Hu , J. Wang , Y. Chen , and L. X. Lai . 2017. “The Comparative Analysis of Acceptance Commitment Therapy and Taoist Cognitive Therapy.” Psychological Techniques and Applications 5, no. 7: 427–432. 10.16842/j.cnki.issn2095-5588.2017.07.006.

[pchj825-bib-0035] Hjemdal, O. , P. A. Vogel , S. Solem , K. Hagen , and T. C. Stiles . 2011. “The Relationship Between Resilience and Levels of Anxiety, Depression, and Obsessive‐Compulsive Symptoms in Adolescents.” Clinical Psychology and Psychotherapy 18, no. 4: 314–321. 10.1002/cpp.719. 20806419

[pchj825-bib-0036] Hou, Y. , and Y. Zhao . 2023. “The ‘Involution’ and ‘Confusion’ of Postgraduate Entrance Exams: An Analysis and Adjustment of College Students' Learning Motivation.” Youth Journal 2: 82–87.

[pchj825-bib-0037] Howell, A. J. , and K. M. Demuynck . 2021. “Psychological Flexibility and Psychological Inflexibility Are Independently Associated With Both Hedonic and Eudaimonic Well‐Being.” Journal of Contextual Behavioral Science 20: 163–171. 10.1016/j.jcbs.2021.04.002.

[pchj825-bib-0038] Howell, A. J. , and H. A. Passmore . 2019. “Acceptance and Commitment Training (ACT) as a Positive Psychological Intervention: A Systematic Review and Initial Meta‐Analysis Regarding ACT's Role in Well‐Being Promotion Among University Students.” Journal of Happiness Studies 20: 1995–2010. 10.1007/s10902-018-0027-7.

[pchj825-bib-0078] Husky, M. M. , V. Kovess‐Masfety , and J. D. Swendsen . 2020. “Stress and Anxiety Among University Students in France During Covid‐19 Mandatory Confinement.” Comprehensive Psychiatry 102: 152191. 10.1016/j.comppsych.2020.152191. 32688023 PMC7354849

[pchj825-bib-0039] Kashdan, T. B. , D. J. Disabato , F. R. Goodman , J. D. Doorley , and P. E. McKnight . 2020. “Understanding Psychological Flexibility: A Multimethod Exploration of Pursuing Valued Goals Despite the Presence of Distress.” Psychological Assessment 32, no. 9: 829–850. 10.1037/pas0000834. 32614192

[pchj825-bib-0040] Kashdan, T. B. , and J. Rottenberg . 2010. “Psychological Flexibility as a Fundamental Aspect of Health.” Clinical Psychology Review 30, no. 7: 865–878. 10.1016/j.cpr.2010.03.001. 21151705 PMC2998793

[pchj825-bib-0041] King, E. L. , C. M. Spencer , and C. A. Meeks . 2021. “How the COVID‐19 Pandemic Can and Must Expand Social Worker e‐Interventions for Mental Health, Family Wellness, and Beyond.” Social Work 67, no. 1: 69–78. 10.1093/sw/swab043. PMC8574323 34694398

[pchj825-bib-0042] Knoop, H. , K. Van Kessel , and R. Moss‐Morris . 2012. “Which Cognitions and Behaviors Mediate the Positive Effect of Cognitive Behavioral Therapy on Fatigue in Patients With Multiple Sclerosis?” Psychological Medicine 42, no. 1: 205–213. 10.1017/S0033291711000924. 21672300

[pchj825-bib-0043] Kruger, L. J. , C. Li , E. Kimble , R. Ruah , D. Stoianov , and K. Krishnan . 2016. “Impact of Repeatedly Failing a High School Exit Exam: Voices of English Language Learners.” Urban Review 48, no. 3: 463–483. 10.1007/s11256-016-0363-z.

[pchj825-bib-0044] Kumle, L. , M. L.‐H. Võ , and D. Draschkow . 2021. “Estimating Power in (Generalized) Linear Mixed Models: An Open Introduction and Tutorial in R.” Behavior Research Methods 53, no. 6: 2528–2543. 10.3758/s13428-021-01546-0. 33954914 PMC8613146

[pchj825-bib-0045] Lakens, D. 2013. “Calculating and Reporting Effect Sizes to Facilitate Cumulative Science: A Practical Primer for *t* ‐Tests and ANOVAs.” Frontiers in Psychology 4: 863. 10.3389/fpsyg.2013.00863. 24324449 PMC3840331

[pchj825-bib-0046] Lam, C. Y. 2017. “The Hope Experience of Young Adults Who Fail in Public Examination.” Asia Pacific Journal of Counselling and Psychotherapy 8, no. 2: 131–149. 10.1080/21507686.2017.1348369.

[pchj825-bib-0047] Landi, G. , K. I. Pakenham , E. Crocetti , S. Grandi , and E. Tossani . 2021. “The Multidimensional Psychological Flexibility Inventory (MPFI): Discriminant Validity of Psychological Flexibility With Distress.” Journal of Contextual Behavioral Science 21: 22–29. 10.1016/j.jcbs.2021.05.004.

[pchj825-bib-0048] Li, C. 2020. “Employment of College Students Under the Impact of the Pandemic: Employment Pressure, Psychological Pressure, and Changes in Employment Choices.” Educational Research 7: 4–16.

[pchj825-bib-0079] Lin, Y. Y. , R. D. Rogge , and D. P. Swanson . 2020. “Cross‐Cultural Flexibility: Validation of the Traditional Mandarin, Simplified Mandarin, and Japanese Translations of the Multidimensional Psychological Flexibility Inventory.” Journal of Contextual Behavioral Science 15: 73–84. 10.1016/j.jcbs.2019.11.008.

[pchj825-bib-0049] Liu, J. J. , N. Ein , J. Gervasio , M. Battaion , M. Reed , and K. Vickers . 2020. “Comprehensive Meta‐Analysis of Resilience Interventions.” Clinical Psychology Review 82: 101919. 10.1016/j.cpr.2020.101919. 33045528

[pchj825-bib-0050] Lovibond, S. H. , P. F. Lovibond , and Psychology Foundation of Australia . 1995. Manual for the Depression Anxiety Stress Scales. 2nd ed. Australia: Psychology Foundation of Australia.

[pchj825-bib-0051] Masten, A. S. 2001. “Ordinary Magic: Resilience Processes in Development.” American Psychologist 56, no. 3: 227–238. 10.1037/0003-066X.56.3.227. 11315249

[pchj825-bib-0052] Masten, A. S. , J. Cutuli , J. E. Herbers , and M.‐G. J. Reed . 2009. “Resilience in Development.” In The Oxford Handbook of Positive Psychology, edited by S. J. Lopez and C. Snyder , 2nd ed. New York, USA: Oxford University Press.

[pchj825-bib-0054] Ministry of Education of the People's Republic of China . 2021. “The Ministry of Education: 2022 College Graduates Are Expected to Be 10.76 Million, an Increase of 1.67 Million.” http://www.moe.gov.cn/fbh/live/2021/53931/mtbd/202112/t20211229_591046.html.

[pchj825-bib-0055] Morris, L. , and W. Mansell . 2018. “A Systematic Review of the Relationship Between Rigidity/Flexibility and Transdiagnostic Cognitive and Behavioral Processes That Maintain Psychopathology. Journal of Experimental.” Psychopathology 9, no. 3: 2043808718779431. 10.1177/2043808718779431.

[pchj825-bib-0056] Murrell, A. R. , R. Jackson , E. G. Lester , and T. Hulsey . 2018. “Psychological Flexibility and Resilience in Parentally Bereaved College Students.” OMEGA‐Journal of Death and Dying 76, no. 3: 207–226. 10.1177/0030222817693154. 28198653

[pchj825-bib-0057] National Bureau of Statistics (NBS) . 2023. “Statistical Communiqué of the People's Republic of China on the 2022 National Economic and Social Development.” http://www.stats.gov.cn/english/PressRelease/202302/t20230227_1918979.html.

[pchj825-bib-0058] Osman, A. , C. L. Bagge , P. M. Gutierrez , L. C. Konick , B. A. Kopper , and F. X. Barrios . 2001. “The Suicidal Behaviors Questionnaire‐Revised (SBQ‐R): Validation With Clinical and Nonclinical Samples.” Assessment 8, no. 4: 443–454. 10.1177/107319110100800409. 11785588

[pchj825-bib-0080] Pakenham, K. I. 2015. “Effects of Acceptance and Commitment Therapy (ACT) Training on Clinical Psychology Trainee Stress, Therapist Skills and Atributes, and ACT Processes.” Clinical Psychology and Psychotherapy 22, no. 6: 647–655. 10.1002/cpp.1924. 25307059

[pchj825-bib-0059] Pakenham, K. I. , M. Mawdsley , F. L. Brown , and N. W. Burton . 2018. “Pilot Evaluation of a Resilience Training Program for People With Multiple Sclerosis.” Rehabilitation Psychology 63, no. 1: 29–42. 10.1037/rep0000167. 29154558

[pchj825-bib-0060] Pan, J.‐Y. 2011. “A Resilience‐Based and Meaning‐Oriented Model of Acculturation: A Sample of Mainland Chinese Postgraduate Students in Hong Kong.” International Journal of Intercultural Relations 35, no. 5: 592–603. 10.1016/j.ijintrel.2011.02.009.

[pchj825-bib-0061] Rogge, R. D. , J. S. Daks , B. A. Dubler , and K. J. Saint . 2019. “It's all About the Process: Examining the Convergent Validity, Conceptual Coverage, Unique Predictive Validity, and Clinical Utility of ACT Process Measures.” Journal of Contextual Behavioral Science 14: 90–102. 10.1016/j.jcbs.2019.10.001.

[pchj825-bib-0062] Rolffs, J. L. , R. D. Rogge , and K. G. Wilson . 2018. “Disentangling Components of Flexibility via the Hexaflex Model: Development and Validation of the Multidimensional Psychological Flexibility Inventory (MPFI).” Assessment 25: 458–482. 10.1177/1073191116645905. 27152011

[pchj825-bib-0081] Son, C. , S. Hegde , A. Smith , X. Wang , and F. Sasangohar . 2020. “Effects of COVID‐19 on College Students’ Mental Health in the United States: Interview Survey Study.” Journal of Medical Internet Research 22, no. 9: e21279. 10.2196/21279. 32805704 PMC7473764

[pchj825-bib-0063] Southwick, S. M. , and D. S. Charney . 2012. “The Science of Resilience: Implications for the Prevention and Treatment of Depression.” Science 338, no. 6103: 79–82. 10.1126/science.1222942. 23042887

[pchj825-bib-0064] Stabbe, O. K. , J. L. Rolffs , and R. D. Rogge . 2019. “Flexibly and/or Inflexibly Embracing Life: Identifying Fundamental Approaches to Life With Latent Profile Analyses on the Dimensions of the Hexaflex Model.” Journal of Contextual Behavioral Science 12: 106–118. 10.1016/j.jcbs.2019.03.003.

[pchj825-bib-0065] Steinhardt, M. , and C. Dolbier . 2008. “Evaluation of a Resilience Intervention to Enhance Coping Strategies and Protective Factors and Decrease Symptomatology.” Journal of American College Health 56, no. 4: 445–453. 10.3200/JACH.56.44.445-454. 18316290

[pchj825-bib-0066] Sun, J. 2023. “Factors Influencing College Students' Career Choice in the Post‐Pandemic Era and Positive Guidance: A Perspective Based on Social Ecological Systems Theory.” Beijing Youth Research 32, no. 1: 60–68.

[pchj825-bib-0067] Tavakoli, N. , A. Broyles , E. K. Reid , J. R. Sandoval , and V. Correa‐Fernández . 2019. “Psychological Inflexibility as It Relates to Stress, Worry, Generalized Anxiety, and Somatization in an Ethnically Diverse Sample of College Students.” Journal of Contextual Behavioral Science 11: 1–5. 10.1016/j.jcbs.2018.11.001.

[pchj825-bib-0068] Thompson, E. M. , L. Destree , L. Albertella , and L. F. Fontenelle . 2021. “Internet‐Based Acceptance and Commitment Therapy: A Transdiagnostic Systematic Review and Meta‐Analysis for Mental Health Outcomes.” Behavior Therapy 52, no. 2: 492–507. 10.1016/j.beth.2020.07.002. 33622516

[pchj825-bib-0070] Vahabi, M. , J. Pui‐Hing Wong , M. Moosapoor , A. Akbarian , and K. Fung . 2022. “Effects of Acceptance and Commitment Therapy (ACT) on Mental Health and Resiliency of Migrant Live‐In Caregivers in Canada: Pilot Randomized Wait List Controlled Trial.” JMIR Formative Research 6, no. 1: e32136. 10.2196/32136. 35084337 PMC9090443

[pchj825-bib-0071] Vauclair, C. M. , R. Fischer , M. C. Ferreira , et al. 2015. “What Kinds of Value Motives Guide People in Their Moral Attitudes? The Role of Personal and Prescriptive Values at the Culture Level and Individual Level.” Journal of Cross‐Cultural Psychology 46, no. 2: 211–228. 10.1177/0022022114557487.

[pchj825-bib-0072] Wang, X. L. 2022. “A Case Explores of Rational‐Emotive Therapy to Alleviate the Psychological Problems of Unsuccessful Students in Postgraduate Entrance Examination,” Master's Dissertation, Gansu Political Science and Law Institute. https://kns.cnki.net/KCMS/detail/detail.aspx?dbname=CMFDTEMP&filename=1022547947.nh.

[pchj825-bib-0073] Waters, C. S. , N. Frude , P. E. Flaxman , and J. Boyd . 2018. “Acceptance and Commitment Therapy (ACT) for Clinically Distressed Health Care Workers: Waitlist Controlled Evaluation of an ACT Workshop in a Routine Practice Setting.” British Journal of Clinical Psychology 57, no. 1: 82–98. 10.1111/bjc.12155. 28857254

[pchj825-bib-0074] Windle, G. , K. M. Bennett , and J. Noyes . 2011. “A Methodological Review of Resilience Measurement Scales.” Health and Quality of Life Outcomes 9, no. 1: 8. 10.1186/1477-7525-9-8. 21294858 PMC3042897

[pchj825-bib-0075] Xu, L. M. 2014. “An Investigation on the Psychological Status of Students Who Failed in Postgraduate Entrance Examination.” Policy Research and Exploration 3: 36–37.

[pchj825-bib-0076] Zhang, D. M. , M. Xiong , and Y. Z. Li . 2018. “The Reliability and Validity of 10‐Item Connor‐Davidson Resilience Scale in the Community‐Dwelling Older Adults.” Chinese Journal of Behavioral Medicine and Brain Science 27, no. 10: 942–946.

