# Supplementary material for: Effect and Mechanism of an ACT‐Based Psychological Resilience Intervention Targeting Students Failing in Postgraduate Entrance Examinations in China: A Randomized Controlled Trial
Source: Psych J. 2025 Jan 8;14(3):344–56. doi: 10.1002/pchj.825 (PMC12133240; doi:10.1002/pchj.825)
Supplement: Supplementary file 1 — Data S1. [file PCHJ-14-344-s001.docx]

**Supplementary Material**

Appendix A

Table A.1

Intervention satisfaction questionnaire

| (a) How do you think the group course works? |
| --- |
| (b) What are your main gains from the group course? |
| (c) What do you think of the necessity of developing group courses for students who fail the postgraduate entrance examination? |
| (d) When and in what form would be appropriate for future groups? |

Table A.2

| 1= very inconsistent to 5= very consistent |
| --- |
| 1. Now I am more aware of my values (or what is important to me) |
| 2. I am more able to act in accordance with my values |
| 3. I can more consciously promote my mental health through the practice |
| 4. I gained more support from my peers |
| 5. I am more capable of providing help to my peers |
| 6. I can talk more openly with others about the topics related to the examination |
| 7. I will be more confident to deal with problems like failure in the future |

Self-assessment questionnaire

**Acceptability**

The attitude of participants on the program was positive. (a) All participants rated the group intervention as effective. (b) 25% of the participants evaluated that their ability was improved, and the goal was more explicit; 60% indicated that they mastered more skills of self-understanding and self-regulation. (c) All participants appraised that the group intervention was very necessary. (d) Most participants (75%) could accept the online intervention but thought it was better to combine offline. For time arrangement, most of them (85%) thought that the time selected for this program was appropriate. Meanwhile, some people (15%) hoped to arrange corresponding guidance when the results of the postgraduate entrance examination came out. Regarding the personal status after the intervention, the scores of participants on the issues of “value clarity”(*M*=3.95; *SD*=.51), “value compliance”(*M*=3.90; *SD*=.55), “conscious practice”(*M*=4.15; *SD*=.36), “coping with failure”(*M*=3.90; *SD*=.72), “discussion failure”(*M*=4.05; *SD*=.61), “getting support”(*M*=4.20; *SD*=.62) and “helping peers”(*M*=3.95; *SD*=.69) were all around four points, which indicated that the intervention had brought them obvious positive changes. No harm was reported in the intervention.

**Drop-out analysis**

There were 67 persons eligible for the program, and 61 persons attended it. During the program, 3 of the intervention group dropped out because of the dissertation, job-hunting and uncompleted post-assessment, and 5 of the control group dropped out because of job search, civil service exams and teacher recruitment exams. The dropout rates were 9.7%, 6.7% for the intervention and control groups respectively (*χ^2^* = 0.184, *df* = 1, *p* = .668). In the 1-month follow-up period, the dropout rates were 9.7%, 16.7% respectively in the two groups (*χ^2^* = 0.654, *df* = 1, *p* = .419). The retention rate was 87% from enrollment to the 1-month follow-up. In terms of measured variables, completers and non-completers did not show substantive differences at baseline.

Appendix B

Table B.1

Overview of The Resilience Group Course

| Topic | ACT process | Content |
| --- | --- | --- |
| 1. Introduction |  | Introduce the program, warm up, and inform the group rules.   - Outline of the program - Get to know each other and establish group rules - Psychoeducation for exam failure and psychological distress - Psychoeducation on resilience and related protective factors - Homework: your expectations |
| 2. Look back to the examination | Acceptance | Review the failure and understand how experiential avoidance affects life.   - Mindfulness breathing - White bear experiment - Looking back on the road to the exam - Another way of thinking - Homework: Healing Hands |
| 3. The brain is talking | Defusion | Experience cognitive fusion and defusion, and understand the meaning of defusion.   - Mindfulness exercise: Fallen leaves floating with the stream - Brain this guy - My brain says - Knowledge explanation: introduce the common mistakes of defusion - Homework: look at the brain from a distance |
| 4. Mindful life | Contact With the Present Moment | Experience mindfulness and apply it in your life.   - Recognize mindfulness - Self-test of mindfulness level - Mindfulness in traditional Chinese exercise - Homework: mindfulness in life |
| 5. Look at yourself differently | Self-as-Context | Reconnect with ourselves and make room for thoughts and feelings.   - Mindfulness exercise: feel the smell of breathing - Plasticine Creation: me after the postgraduate entrance examination - Look at myself anew - Homework: separate a me |
| 6. Value | Values | Clarify the differences between values and goals and define values.   - Mindfulness exercise: the power of self-care - Are my values my own? - Knowledge explanation: values are different from goals - Homework: value self-test form |
| 7. My Future, my choice | Commitment | Develop action plans based on values and anticipate possible difficulties and solutions.   - Mindfulness exercise: mindful facing thoughts - The goal walks with me - Refinement of the goal - My support system - Homework: mini plan |
| 8. Let us go again |  | Review the courses, reinforce the harvest and support, and look to the future.   - Mindfulness Exercise: breathing exercise - Looking back at yesterday: share the harvest - A confession of love: appreciate each other and build further mutual support - Post-test |

Appendix C

**The Delphi expert consultation**

The Delphi expert consultation is a decision-making method based on expert consensus. Eight experts with extensive experience in group counseling and/or proficiency in ACT were invited to seek their opinions for this study. The first round of expert consultation focused on soliciting expert opinions on the soundness of the initially proposed group program. The main components include topic selection and activity composition. The consultation was conducted through face-to-face or email communication and clarification of the purpose and significance of the study. After the first round of expert consultation questionnaires were all returned, the researchers compiled and statistically processed them according to the expert opinions. Then the research group discussed and revised the corresponding entries, along with feedback on the first round of experts' comments (e.g., reasons for adoption or non-adoption) to form a second round of expert consultation questionnaire. Experts were asked to give further modifications with reference to the feedback information. Finally, the final intervention plan was formed after two rounds of expert consultation.

The expert consultation questionnaire consisted of two parts, one was the basic expert questionnaire, which included personal information of the expert, the basis of the evaluation of the program entries, familiarity with the study content, and overall evaluation of the study. The second was the evaluation of the specific content of the intervention program, which used a Likert 5-point scale to make judgments and choices about the importance of the entries and to give individual suggestions for modifications. The evaluation indexes of the degree of expert authority (Authority Coefficient) and the degree of expert opinion coordination (Coefficient of Variation) were finally obtained by statistical analysis. It is generally considered that the results of expert authority coefficient≥0.7 and variation coefficient<0.25 are acceptable (Diamond et al., 2014).

Table C.1

Concentration and Coefficient of Variation of the Overall Evaluation of the Intervention Program in Two Rounds.

|  | Round 1 | | | Round 2 | | |
| --- | --- | --- | --- | --- | --- | --- |
| program | *M* | *SD* | *CV(M/SD)* | *M* | *SD* | *CV(M/SD)* |
| scientificity | 3 | 0 | 0 | 2.87 | 0.35 | 0.12 |
| Operability | 2.75 | 0.46 | 0.17 | 3 | 0 | 0 |
| goal clarity | 2.87 | 0.35 | 0.12 | 3 | 0 | 0 |
| Structural rationality | 2.75 | 0.46 | 0.17 | 3 | 0 | 0 |

Notes:*M*=mean; *SD*=standard deviation; *CV*=variation coefficient

Appendix D

Table D.1

Observed and Estimated Means of Six Dimension of PI and Within- and Between-group Effect Sizes

|  | Pre-assessment | *n* | Post-assessment | *n* | Follow up | *n* | Pre-post within | Post between | Pre-follow-up within | Follow-up between |
| --- | --- | --- | --- | --- | --- | --- | --- | --- | --- | --- |
|  | *M (SD)* |  | (estimated)  *M (SE)* |  | (estimated)  *M (SE)* |  | *d_cohen’s_(CI)* | *d_cohen’s_(CI)* | *d_cohen’s_(CI)* | *d_cohen’s_(CI)* |
| Experiential Avoidance |  |  |  |  |  |  |  |  |  |  |
| IG | 6(2.39) | 31 | 6.75(0.494) | 31 | 6.822(0.383) | 31 | 0.33(-0.17,0.83) | 0.38(-0.12,0.89) | 0.41(-0.08,0.92) | -0.09(-0.59,0.41) |
| CG | 6.9(1.76) | 30 | 5.717(0.495) | 30 | 7.013(0.402) | 30 | -0.49(-1.01,0.01) |  | 0.02(-0.49,0.52) |  |
| Fusion |  |  |  |  |  |  |  |  |  |  |
| IG | 4.87(2.50) | 31 | 4.48(0.50) | 31 | 4.33(0.47) | 31 | -0.12(-0.62,0.37) | 0.25(-0.25,0.75) | -0.18(-0.68,0.32) | 0.09(-0.40,0.60) |
| CG | 3.87(2.80) | 30 | 3.80(0.50) | 30 | 4.07(0.49) | 30 | -0.02(-0.53,0.48) |  | 0.07(-0.44,0.58) |  |
| Inattentive and Unaware |  |  |  |  |  |  |  |  |  |  |
| IG | 5.97(2.51) | 31 | 4.51(0.49) | 31 | 4.76(0.49) | 31 | **-0.77(-1.28,-0.25)** | **-0.60(-1.11,-0.09)** | -0.41(-0.92,0.09) | -0.08(-0.59,0.41) |
| CG | 4.55(2.47) | 30 | 6.09(0.49) | 30 | 5.00(0.51) | 30 | **0.56(0.05,1.07)** |  | 0.15(-0.36,0.65) |  |
| Self as Content |  |  |  |  |  |  |  |  |  |  |
| IG | 5.3(2.87) | 31 | 3.27(0.50) | 31 | 3.63(0.49) | 31 | **-0.70(-1.21,-0.19)** | **-1.02(-1.55,-0.49)** | **-0.57(-1.08,-0.06)** | -0.30(-0.81,0.20) |
| CG | 4.29(2.69) | 30 | 6.04(0.50) | 30 | 4.21(0.51) | 30 | 0.61(0.09,1.12) |  | -0.02(-0.53,0.49) |  |
| Losing Touch with Values |  |  |  |  |  |  |  |  |  |  |
| IG | 5.27(2.53) | 31 | 4.51(0.50) | 31 | 4.79(0.50) | 31 | **-0.53(-1.03,-0.02)** | -0.43(-0.94,0.07) | **-0.67(-1.18,-0.16)** | -0.23(-0.74,0.27) |
| CG | 5.35(2.24) | 30 | 5.70(0.51) | 30 | 5.43(0.51) | 30 | -0.21(-0.72,0.30) |  | -0.23(-0.73,0.28) |  |
| Inaction |  |  |  |  |  |  |  |  |  |  |
| IG | 6.6(2.57) | 31 | 3.93(0.52) | 31 | 3.72(0.43) | 31 | **-0.69(-1.10,-0.18)** | -0.29(-0.79,0.21) | **-0.59(-1.10,-0.08)** | -0.30(-0.81,0.20) |
| CG | 5.71(3.38) | 30 | 4.75(0.52) | 30 | 4.46(0.46) | 30 | -0.01(-0.51,0.49) |  | -0.10(-0.61,0.41) |  |

Note:IG=intervention group; CG=control group; *M*=mean; *SD*=standard deviation; *SE*= standard error; *d_cohen’s_* based on estimated means and the pooled standard deviations.

**Reference**

Diamond, I. R., Grant, R. C., Feldman, B. M., Pencharz, P. B., Ling, S. C., Moore, A. M., & Wales, P. W. (2014). Defining consensus: A systematic review recommends methodologic criteria for reporting of Delphi studies.*Journal of clinical epidemiology, 67*(4), 401-409.

[https://doi.org/10.1016/j.jclinepi.2013.12.002](https://doi.org/10.1016/j.jclinepi.2013.12.002).
